# Inclusion of 11-Oxygenated Androgens in a Clinical Routine LC-MS/MS Setup for Steroid Hormone Profiling

**DOI:** 10.3390/ijms24010539

**Published:** 2022-12-29

**Authors:** Robert Zeidler, Ronald Biemann, Uta Ceglarek, Jürgen Kratzsch, Berend Isermann, Alexander Gaudl

**Affiliations:** Institute of Laboratory Medicine, Clinical Chemistry and Molecular Diagnostics, Leipzig University, Liebigstraße 27, 04103 Leipzig, Germany

**Keywords:** 11-oxygenated androgens, androgens, steroid hormones, LC-MS/MS, method validation

## Abstract

11-Oxygenated androgens (11-OAs) are being discussed as potential biomarkers in diagnosis and therapy control of disorders with androgen excess such as congenital adrenal hyperplasia and polycystic ovary syndrome. However, quantification of 11-OAs by liquid chromatography-tandem mass spectrometry (LC-MS/MS) still relies on extensive sample preparation including liquid–liquid extraction, derivatization and partial long runtimes, which is unsuitable for high-throughput analysis under routine laboratory settings. For the first time, an established online-solid-phase extraction-LC-MS/MS (online-SPE-LC-MS/MS) method for the quantitation of seven serum steroids in daily routine use was extended and validated to include 11-ketoandrostenedione, 11-ketotestosterone, 11β-hydroxyandrostenedione and 11β-hydroxytestosterone. Combining a simple protein precipitation step with fast chromatographic separation and ammonium fluoride-modified ionization resulted in a high-throughput method (6.6 min run time) featuring lower limits of quantification well below endogenous ranges (63–320 pmol/L) with recoveries between 85% and 117% (CVs ≤ 15%). Furthermore, the ability of this method to distinguish between adrenal and gonadal androgens was shown by comparing 11-OAs in patients with hyperandrogenemia to healthy controls. Due to the single shot multiplex design of the method, potential clinically relevant ratios of 11-OAs and corresponding androgens were readily available. The fully validated method covering endogenous concentration levels is ready to investigate the diagnostic values of 11-OAs in prospective studies and clinical applications.

## 1. Introduction

**Figure 1:**
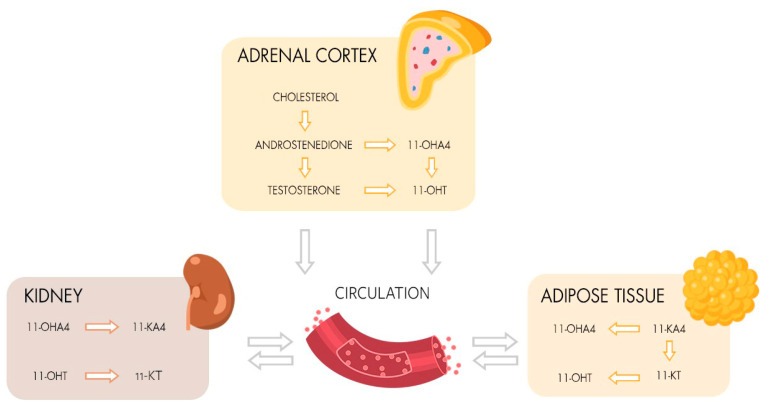
Metabolism of 11-OAs and their circulation in peripheral tissues. Circulating 11-OHA4 and 11-OHT is de novo synthesized from cholesterol via androstenedione and testosterone in adrenal glands. 11-OHA4 and 11-OHT are metabolized to 11-KA4 and 11-KT in kidneys and vice versa converted as well as metabolized to 11-KA4 from 11-KT in adipose tissues. 11-KA4, 11-ketoandrostenedione; 11-KT, 11-ketotestosterone; 11-OHA4, 11β-hydroxyandrostenedione; 11-OHT, 11β-hydroxytestosterone.

The adrenal gland is the source of 11-hydroxylated androgens, which are primary synthesized in the *zona reticularis* by 11β-hydroxylase (CYP11B1) from androstenedione and testosterone under regulation of adrenocorticotropic hormone [1]. The low potent androgens 11β-hydroxyandrostenedione (11-OHA4) and 11β-hydroxytestosterone (11-OHT) are precursors for the higher potent 11-ketoandrostenedione (11-KA4) and 11-ketotestosterone (11-KT) which are formed by 11β-HSDB2 (11β-hydroxysteroid dehydrogenase type 2) in adrenal glands and kidneys (Figure 1) [2,3,4,5]. In adipose tissues, the synthesis of 11-KT from 11-KA, as well as the metabolization of the higher potent 11-ketoandrogens to 11-hydroxylated androgens by 11β-HSDB1 (11β-hydroxysteroid dehydrogenase type 1), is suggested [3,6,7]. Due to their origin, 11-oxygenated androgens (11-OAs) allow the differentiation of adrenal- and gonadal-produced androgens and provide a potential diagnostic tool to reliably assign contributions of these organs to disorders of androgen synthesis, such as polycystic ovarian syndrome (PCOS), androgen-producing or -dependent tumors, and for therapy control in congenital adrenal hyperplasia (CAH) [3,8,9,10,11,12,13,14]. Furthermore, the ratios of 11-OAs to testosterone or androstenedione are discussed as potential biomarkers regarding disorders of androgen synthesis [12,13,15]. 

Considering their diagnostic potential, 11-OAs are not yet established as parameters in clinical routine analysis, due to low serum concentration levels, high laborious effort and interferences in routine immunoassays. First mass spectrometry-based methods to reliably quantify 11-OAs were published between 2008 and 2011 and were limited by, e.g., lack of corresponding internal standards, long runtimes, low sensitivity or missing validation for human serum samples as well as a lack of certified calibrators and quality controls [16,17,18,19]. In recent years, several newly developed LC-MS/MS methods have been published partially overcoming those limitations [10,13,15,20,21,22,23,24,25,26,27,28,29,30,31,32]. These analytical methods, including validation data as well as advantages and disadvantages, are described in detail in Caron et al. (2021) [21]. Overall, published methods rely on extensive sample preparation including liquid–liquid extraction and derivatization, which is unsuitable for high-throughput analysis in clinical routine diagnostics. Therefore, the major objective of this work was the inclusion of 11-oxygenated androgens in an established routine online-SPE-LC-MS/MS setup for profiling of seven clinically relevant steroid hormones including 17α-hydroxyprogesterone (17-OHP), aldosterone (A), androstenedione (A4), cortisol (F), cortisone (E), dehydroepiandrosterone sulfate (DHEAS), estradiol (E2), progesterone (P) and testosterone (T). Details of the method are described in Gaudl et al., (2016) [33].

## 2. Results

**Figure 2:**
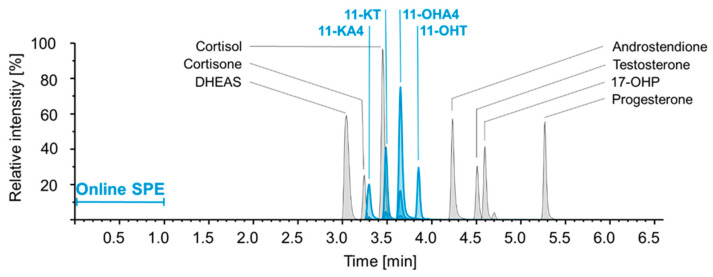
Exemplary chromatogram for single shot analysis of 11 steroid hormones. Chromatographic separation with retention times and relative intensities of 11-OAs (blue; 11-KA4, 11-KT, 11-OHA4 and 11-OHT) and established steroid panel (grey; 17-OHP, androstenedione, cortisol, cortisone, DHEAS, testosterone and progesterone) [34]. 11-KA4, 11-ketoandrostenedione; 11-KT, 11-ketotestosterone; 11-OHA4, 11β-hydroxyandrostenedione; 11-OHT, 11β-hydroxytestosterone; 17-OHP, 17α-hydroxyprogesterone; DHEAS, dehydroepiandrosterone sulfate; SPE, solid phase extraction.

Multiple reaction monitoring of the four 11-OAs was integrated into the established LC-MS/MS setup as shown in Figure 2. Limits of detection (LODs) were calculated at 15 pmol/L for 11-KA4, 18 pmol/L for 11-KT, 32 pmol/L for 11-OHA4 and 19 pmol/L for 11-OHT (see Table S2). Linearity was proven between 0.08 and 3.3 nmol/l for 11-KA4, 11-KT, 11-OHT, and between 0.8 and 33 nmol/l for 11-OHA4. Relative standard deviations of the slopes of the calibration curves were below 4% with R² > 0.999. Among the 11-OAs, multiple interferences were observed (Table S1). Interferences above 1% of the original signal intensity were chromatographically separated (R > 1.5) except for 11-OHA4-d7 interfering with 11-OHT, adding 1.3% of its original signal intensity to the analyte. In serum samples, post column infusion showed an intensity loss by the first fraction of matrix constituents reaching the mass spectrometer and affected all analytes starting with the red dashed line in example chromatograms for 11-OAs in Figure S1. The ion suppression by the matrix of serum samples was observed across the detection window affecting all mass transitions of all analytes and internal standards (intensity loss in serum: 40% for 11-KA4, 50% for 11-KT and 11-OHA4 and 30% for 11-OHT). Specific ion-suppressing effects at the retention times of the analytes were not detected. Example chromatograms are shown in Figure S1.

**Figure 3:**
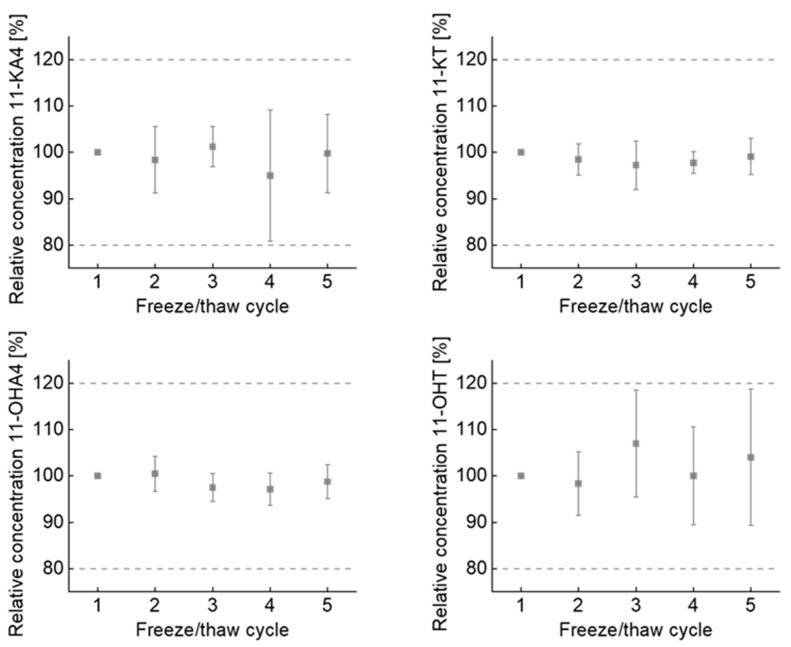
11-OAs are stable across repeated freeze/thaw cycles. Dot plots show mean and coefficient of variation of 5 individually sample preparations. Data are presented relative to the concentration of first freeze/thaw cycle. Analyte ranges were from 3.1 nmol/L to 8.1 nmol/L for 11-OHA4, 0.3 nmol/L to 0.8 nmol/L for 11-KA4, 0.3 nmol/L to 1.2 nmol/L for 11-OHT and 0.6 nmol/L to 2.6 nmol/L for 11-KT. Error bars represent standard derivations, dashed lines indicate the acceptable limit of change (±20%). 11-KA4, 11-ketoandrostenedione; 11-KT, 11-ketotestosterone; 11-OHA4, 11β-hydroxyandrostenedione; 11-OHT, 11β-hydroxytestosterone;.

Inter- and intra-assay coefficients of variation (CVs) in spiked serum and spiked controls were between 2% and 13% for 11-KA4, 2% and 15% for 11-KT, 2% and 7% for 11-OHA4, 2% and 10% for 11-OHT. Mean recovery ranges in spiked serum and spiked controls were between 102% and 115% for 11-KA4, 85% and 105% for 11-KT, 100% and 114% for 11-OHA4 and 99% and 117% for 11-OHT (Table 1). Lower limits of quantification (LLOQs) were determined at 63 pmol/L for 11-KA4 (CV 20%, *s/n* = 13), 100 pmol/L for 11-KT (CV 9%, *s/n* = 17), 320 pmol/L for 11-OHA4 (CV 3.9%, *s/n* = 30) and 83 pmol/L for 11-OHT (CV 5.7%, *s/n* = 13). In freeze/thaw stability experiments, the concentrations of 11-OAs were stable across five cycles with a reproducibility within the acceptable limit of 20% and without increasing or decreasing trend (Figure 3). 

**Figure 4:**
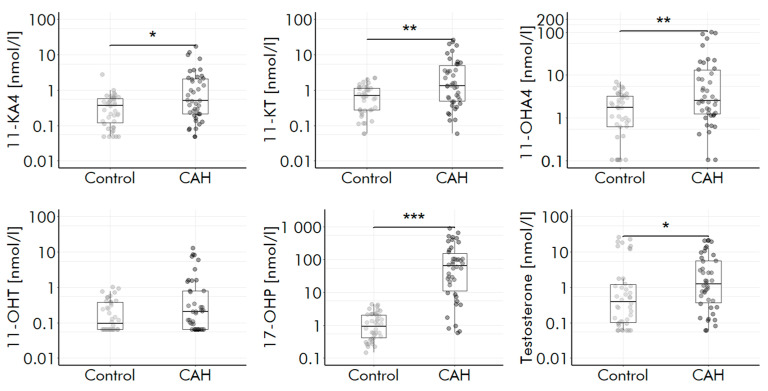
Levels of 17-OHP, testosterone and 11-OAs are elevated in CAH. Boxplots show median and interquartile range, whiskers indicate 95% confidence interval; * *p* < 0.01, ** *p* <0.05, *** *p* < 0.001. CAH, congential adrenal hyperplasia; 11-KA4, 11-ketoandrostenedione; 11-KT, 11-ketotestosterone; 11-OHA4, 11β-hydroxyandrostenedione; 11-OHT, 11β-hydroxytestosterone; 17-OHP, 17α-hydroxyprogesterone.

For clinical verification, comparison of CAH patients and healthy controls revealed elevated levels of 11-KA4 (*p* = 0.016), 11-OHA4 (*p* = 0.006), 11-OHT (*p* = 0.08), 11-KT (*p* = 0.001), 17 OHP (*p* < 0.001), T (*p* = 0.015) and DHEAS (*p* = 0.012) as expected (Figure 4 and Table S6). 

Accordingly, ratios of A/11-OHT (*p* = 0.004), DHEAS/11-OAs (*p* < 0.001), T/11-OHT (*p* = 0.042) were decreased in CAH patients, reflecting a higher adrenal versus gonadal androgen synthesis. 11-OA levels in the 42 healthy individuals were comparable to published concentration levels for 11-OAs (Table S3).

## 3. Discussion

11-OAs are currently being discussed as biomarkers to improve diagnosis and therapy of diseases that are associated with disturbed androgen production, including CAH, PCOS, premature adrenarche, metabolic syndrome or obesity [3,8,9,10,11,12,13,14]. However, the quantification of 11-OA is labor intensive and remains a challenge for routine clinical applications. By implementing 11-OAs into an established routine online solid phase extraction online-SPE-LC-MS/MS setup for steroids, these challenges were already resolved and we enabled the simultaneous analysis of 11-OAs and seven relevant steroids within a single run using only 100 µL serum. The presented method covers 11 clinically relevant steroid hormones with fast and simple sample preparation protocol and a short runtime of 6.6 min. Compared to run times of published high-throughput LC-MS/MS methods (4 to 15 min) the presented method can be ranked within the fast ones [14,15,20,23,25,27,28,30]. While these methods utilize liquid–liquid extraction, including evaporation and re-suspension, the single protein precipitation step of the described method generates minimal hands-on time prior to LC-MS/MS measurement, which has been proven to be reliable during five years of routine application [33,34].

Similar to the referenced routine steroid hormones method, imprecision as well as recoveries are within the acceptable limits given by CLSI guideline C62-A, confirming the validity of the method [35]. The obtained sensitivity competes with the most sensitive methods utilizing complex derivatization and multi-stage liquid–liquid extraction [10,24,27,30,32]. In contrast to others, LLOQs and LODs were determined in native sera, reflecting genuine patient samples instead of diluted standards or serum with decreasing matrix influence. Since the experimentally determined LLOQs of the current method are at least three times below the published endogenous ranges of 11-OAs (Table S3), the proposed method is sufficiently sensitive to improve the assessment of clinically relevant hyperandrogenism [7]. 

Through previous experience in steroid analysis, interferences between the analytes with similar masses ranging between 301.2 g/mol and 311.2 g/mol and similar fragments were expected (Table S5) as well as interfering masses by incomplete deuteration of internal standards and proton substitution within the ion source. Sufficient chromatographic separated interferences were non-relevant and neglected. The interference of 11-OHA-d7 with 11-OHT delivers an 1.3% of 4e5 intense signal which is negligible below the noise of 2e3 for 11-OHT in endogenous samples.

In post-column experiments, the loss of sensitivity in serum compared to methanol and calibrator is most probably caused by the serum matrix affecting standards and internal standards equally. Therefore, a negative effect on the determined concentration of any given analyte can be neglected. The negative effect on LLOQs, however, is considered as a price of compromising between sensitivity, selectivity, and high-throughput capability. As repeated freeze/thaw cycles (*n* = 5) have no effect on the analytical stability of 11-OAs, the method is also suitable for batchwise analysis of clinical studies. 

To improve clinical diagnostics, the method must reliably discriminate between patients with and without disturbed androgen production. The determined levels for 11-OAs in 13 healthy individuals are comparable to reported concentration levels for 11-OAs in healthy individuals, thus indicating a correct determination of 11-OAs by the method [7,32]. Elevated levels of 11-KA4, 11-KT and 11-OHA4 in CAH patients similar to recently reported findings in adults are proving the clinical verification of the method (Figure 4 and Table S3). The non-significant elevation of 11-OHT indicates a lack of power due to small sample size of *n* = 42. Decreased ratios of DHEAS and T to 11-OAs confirm the expected increased adrenal versus gonadal androgen synthesis in CAH patients [36]. Despite a fast and easy sample preparation protocol combined with short runtimes, the method delivers results for CAH patients as well as healthy individuals that are similar to recently reported methods utilizing liquid–liquid extraction and derivatization. Since commercial calibrators and traceable controls for 11-OAs are not accessible yet, the fully validated method containing in-house spiked calibrators and controls is ready to investigate the diagnostic values of 11-OAs in prospective studies and clinical applications. A minimal sample volume of 100 µL makes it highly relevant for children as well.

In conclusion, we present a robust high-throughput method with high sensitivity for simultaneous quantification of four 11-OAs and seven relevant steroid hormones using minimal sample preparation. By multiplex-design in single shot analysis, ratios of clinically relevant steroids to 11-OAs are instantly accessible, allowing the application of 11-OAs in future routine diagnostics and therapy control in disorders associated with androgen excess. In future studies, the method will be used for determination of reference intervals (from birth to 80 years) as well as to investigate the influence of 11-OAs on obesity, metabolic syndrome, and puberty.

## 4. Materials and Methods

### 4.1. Chemicals and Reagents

Deionized water was produced in-house using a Barnstead Nanopure from Thermo Scientific, Waltham, MA, USA. Zinc sulfate heptahydrate was obtained from Merck, Darmstadt, Germany, ammonium fluoride from Sigma Aldrich, St. Louis, MO, USA, LC-MS grade methanol from Biosolve, Valkenswaard, The Netherlands. 11-KA4, 11-KT, 11-OHA4, 11-OHT were purchased from Steraloids, Inc., Newport, RI, USA, 11-KT-16,16,17-d3 from Eurisotop GmbH, Saarbrücken, Germany, 11-OHT-2,2,4,6,6-d5 from CDN Isotopes, Pointe-Claire, Quebec, Canada, 11-KA4-3,3,6,6,7,7,9,10,10,17-d10 from LGC Group, Luckenwalde, Germany and 11-OHA4-2,2,4,6,6,16,16-d7 from EQ Laboratories GmbH, Augsburg, Germany.

Methanolic working standards were produced for all analytes and internal standards based on 1 mg/mL (3.3 mmol/L) stock solutions. 6PLUS1 Multilevel serum calibrator levels 1–5 as well as MassCheck^®^ steroid serum control levels 1–3 were obtained from Chromsystems Instruments and Chemicals GmbH, Munich, Germany and were used to generate calibrators and quality controls (QC) and serum covering expected endogenous levels by spiking with working standards of 11-KA4, 11-KT, 11-OHA4, 11-OHT (Table S4) [7]. Spiked Calibrators ranged from 0.08 nmol/L to 33.3 nmol/L for 11-KA4, 11-KT and 11-OHT, and from 0.83 nmol/L to 331 nmol/L for 11-OHA4. Spiked QC’s and serum controls ranged from 0.16 nmol/L to 16.6 nmol/L for 11-KA4, 11-KT and 11-OHT, and from 1.7 nmol/L to 165 nmol/L for 11-OHA4 (Table S4). 

### 4.2. Human Samples

Residual serum taken from routine diagnostics of patients with treated CAH was used for method verification. The study was approved by the ethics committee of the University Hospital Leipzig (082 10 190-42010) according to the declaration of Helsinki ethical principles. Serum samples of healthy individuals with normal 17-OHP concentrations were obtained from the LIFE Child study (Leipzig Research Centre for Civilization Diseases) approved by the Ethical Committee of the University of Leipzig (reference number: Reg. No. 264-10-19042010) and is registered at ClinicalTrials.gov (NCT02550236).

### 4.3. Sample Preparation

Aliquots of calibrators, quality controls, blank, and serum (100 µL) were treated with 200 µL precipitating agent (ZnSO4 in water (0.3 mol/L)/methanol 1/4 *v*/*v*, including the internal standards (3.3 nmol/L)), thoroughly mixed and centrifuged for 10 min at 14,000× *g*. The supernatant was transferred to autosampler vials with 250 µL inserts. 

### 4.4. LC-MS/MS 

A Prominence UFLC system from Shimadzu (Duisburg, Germany) was coupled to a QTRAP^®^ 6500plus from SCIEX (Framingham, MA, USA). A PAL3 RSI autosampler from CTC Analytics (Zwingen, Switzerland) handled sample injection. Injection volume was 100 µL. Online solid phase extraction was performed on a POROS^®^ column (30 × 2.1 mm) from Applied Biosystems (Foster City, CA, USA) at a flow rate of 3 mL/min. For chromatographic separation, a Chromolith^®^ High Resolution column (RP-18, endcapped, 100 × 4.6 mm) from Merck (Darmstadt, Germany) was used. The mobile phase consisted of 50% eluent A (0.2 mmol/L ammonium fluoride (NH4F) in water/methanol 97/3 *v*/*v*) and 50% eluent B (0.2 mmol/L NH4F in water/methanol 3/97 *v*/*v*) and was adjusted as follows: 0–1 min 50% B, 1–5.5 min 50% to 95% B, 5.5–6.5 min 100% B, 6.5–6.6 min 50% B. Flow rate was 1.5 mL/min and the column oven was set to 35 °C. Electrospray ionization (ESI) was applied in positive mode and detection was carried out using multiple reaction monitoring. Mass transitions of the 11-OAs as well as their corresponding internal standards are listed in Table S5. The concentrations were determined using calibration curves which were obtained via ratios of analyte peak area/deuterated standard peak area. Furthermore, estradiol (E2) and aldosterone (A) (ESI negative mode) can additionally be determined without extra sample preparation [33,34].

### 4.5. Validation 

Serum samples with known low concentrations of androgens were used for the determination of LLOQ and LOD. LOD was calculated at *s/n* = 3. LLOQ was defined as the lowest concentration at which a triplicate measurement resulted in CV ≤ 20% with signal to noise (*s/n*) ≥ 10. Linear range of calibration was determined by regression analysis. Means of slopes and regression coefficients of ten 5-point calibrations were determined for robustness of linearity. Potential interferences between 11-OAs, 9 established steroid hormones (17-OHP, A, A4, F, E, E2, DHEAS, T, P), as well as their corresponding internal standards were investigated by measuring highly concentrated standard solutions (28–37 nmol/L; 27 µmol/L for DHEAS/DHEAS-d6). Matrix effects were investigated by post column infusion of 11-OHA4, 11-KA4, 11-OHT and 11-KT (50 nmol/L, 10 µL/min) during the measurement of methanol, calibrator and serum. Imprecision (intra- and inter-assay) and recoveries were determined by measurement of 10 replicates of spiked quality controls as well as spiked serum samples at three concentration levels each as described above. To asses potential effects of freeze/thaw cycles, five serum samples were frozen at 80 °C within 2 h after blood sampling, thawed and refrozen on 4 individual days prior to threefold measurement. Changes above 20% indicated that repeated freeze/thaw cycles affect analytical stability of 11-OAs.

### 4.6. Clinical Verification 

Residual serum samples of 42 patients with treated CAH (19 males, 2 to 79 years) and expected elevated concentrations of 11-OAs were compared to a control group of 42 healthy individuals matched for sex and age. In both groups, concentrations of 11-OAs as well as the routine steroid hormone panel (17-OHP, A, A4, F, E, E2, DHEAS, T, P) and ratios of 17-OHP, A, A4, P or T to 11-OAs were compared.

## Figures and Tables

**Table 1:** Inter-assay imprecision and recovery of 11-oxygenated androgens. Means and coefficients of variation for spiked serum and quality controls at low, moderate and high concentration levels.

	11-KA4			11-KT			11-OHA4			11-OHT		
	Mean [nmol/L]	CV	Recovery	Mean [nmol/L]	CV	Recovery	Mean [nmol/L]	CV	Recovery	Mean [nmol/L]	CV	Recovery
Serum Level 1	0.34	13%	102%	1.5	7%	91%	6.0	5%	113%	0.44	10%	108%
Serum Level 2	0.9	10%	107%	1.9	4%	86%	12	6%	114%	0.62	9%	113%
Serum Level 3	19	7%	115%	16	3%	88%	188	4%	111%	1.2	6%	116%
QK Level 1	0.18	7%	109%	0.14	10%	85%	1.8	5%	107%	0.18	13%	109%
QK Level 2	0.7	10%	108%	0.6	4%	85%	7.1	3%	107%	0.69	5%	105%
QK Level 3	19	8%	113%	15	5%	90%	165	6%	100%	16	3%	99%

QK, quality control; 11-KA4, 11-ketoandrostenedione; 11-KT, 11-ketotestosterone; 11-OHA4, 11β-hydroxyandrostenedione; 11-OHT, 11β-hydroxytestosterone; CV, coefficient of variation.

## Data Availability

Not applicable.

## Supplementary Materials

The supporting information can be downloaded at: https://www.mdpi.com/article/10.3390/ijms24010539/s1.
