# Supplementary material for: Inclusion of 11-Oxygenated Androgens in a Clinical Routine LC-MS/MS Setup for Steroid Hormone Profiling
Source: Int J Mol Sci. 2022 Dec 29;24(1):539. doi: 10.3390/ijms24010539 (PMC9820169; doi:10.3390/ijms24010539)
Supplement: Supplementary file 1 [file ijms-24-00539-s001.zip › ijms-2038524-supplementary.pdf]

Supplement

Table S1. Result overview of interference experiments. Injection of 11-OHA4-d7 delivers a relevant signal (*) with an intensity of 1.3% of its own intensity on the mass trace of 11-OHT. *: Relevant signals with insufficient chromatographic separation.

		Injected analyte										
Mass trace of interfered analyte	Analytes	11-KA4	11-KA4-d10	11-KT	11-KT-d3	11-OHA4	11-OHA4-d7	11-OHT	11-OHT-d5	F	E	DHEAS
	11-KA4	100%		0.12%*		<0.1%*	0.2%*	<0.1%*			0.3%*	
	11-KA4-d10		100%				1.6					0.8%
	11-KT	2.0%		100%		3.4%	0.5%*	<0.1%*				
	11-KT-d3			0.2%*	100%		0.2%*	<0.1%*				
	11-OHA4	1.3%		63%		100%	0.2%*	<0.1%*		0.3%*		
	11-OHA4-d7		0.3%				100%		93%			
	11-OHT			0.5%	1.2%	2%	1.3%*	100%				
	11-OHT-d5		3.9%				79%		100%			
	F									100%		
	E										100%	
	DHEAS											100%

11-KA4, 11-ketoandrostenedione; 11-KT, 11-ketotestosterone; 11-OHA4, 11β-hydroxyandrostenedione; 11-OHT, 11β-hydroxytestosterone; F, cortisol; E, cortisone; DHEAS, dehydroepiandrosterone sulfate.

Table S2. Experimental and calculcated LLOQs as well as calculated LODs for 11-OAs.

**Analyte**	**LLOQ (exp., n=3)**			**LLOQ (calc., *s/n* = 10)**	**LOD (calc., *s/n* = 3)**
	[pmol/l]	CV [%]	s/n	[pmol/l]	[pmol/l]
11-KA4	63	20	13	48	15
11-KT	100	9.1	17	59	18
11-OHA4	320	3.9	30	107	32
11-OHT	83	5.7	13	64	19

11-KA4, 11-ketoandrostenedione; 11-KT, 11-ketotestosterone; 11-OHA4, 11β-hydroxyandrostenedione; 11-OHT, 11β-hydroxytestosterone; CV, coefficient of variation; *s/n*, signal to noise.

Table S3. Means and standard derivation (SD) of determined concentrations of 11-OAs in 42 healthy individuals (19 males) between 2 and 79 years. [a]Mean of median concentrations of 83 females between 4 and 10 years. (Rege 2018, doi: 10.1210/jc.2018-00736) [b]Mean of mean concentrations of 141 healthy individuals (69 males) between 18 and 39 years. (Davio 2020, doi:10.1210/clinem/dgaa343)

	11-KA4 [nmol/l]	11-KT [nmol/l]	11-OHA4 [nmol/l]	11-OHT [nmol/l]
LLOQ	0.063	0.100	0.320	0.083
Mean (SD)	0.41 (0.45)	0.78 (0.59)	2.10 (1.8)	0.25 (0.25)
Mean[a]	0.36	0.44	0.80	0.15
Mean[b]	0.53	0.84	3.98	0.39

Table S4. Concentrations of 17-OHP, A4, F, E, DHEAS, P and T in Chromsystems 6PLUS1 Multilevel serum calibrator level 1-5 and MassCheck® steroid serum control level 1-3 as well as spike concentration of 11-OAs in Chromsystems calibrators, Chromsystems controls and serum controls.

	**Chromsystems 6PLUS1 Multilevel serum calibrator**							**Spike concentrations of 11-OAs in Chromsystems 6PLUS1 Multilevel serum calibrator**			
	17-OHP [nmol/l]	A4 [nmol/l]	F [nmol/l]	E [nmol/l]	DHEAS [nmol/l]	P [nmol/l]	T [nmol/l]	11-KA4 [nmol/l]	11-KT [nmol/l]	11-OHA4 [nmol/l]	11-OHT [nmol/l]
Level 1	0.284	0.646	27.9	2.8	299	0.464	0.194	0.083	0.083	0.83	0.082
Level 2	1.45	1.28	55.7	6.99	1427	2.44	0.884	0.33	0.33	3.3	0.33
Level 3	2.84	2.48	111	13.9	2811	6.39	3.45	0.83	0.83	8.3	0.82
Level 4	5.78	4.78	219	28.6	5547	15.9	10.3	3.3	3.3	33.1	3.3
Level 5	11.5	15.9	403	56.9	10972	31.8	23	33.3	33.1	331	32.8
	**MassCheck® steroid serum control**							**Spike concentrations of 11-OAs in MassCheck® steroid serum control**			
Level 1	0.923	1.01	71.4	5.65	800	0.919	0.669	0.17	0.17	1.7	0.16
Level 2	4.65	4.14	175	34.2	4260	10.1	5.26	0.67	0.66	6.6	0.66
Level 3	27.1	33.5	496	81.5	13600	48.2	27.1	16.6	16.5	165	16.4
								**Spike concentrations of 11-OAs in serum control**			
Level 1								0.17	0.17	1.7	0.16
Level 2								0.67	0.66	6.6	0.66
Level 3								16.6	16.5	165	16.4

17-OHP. 17α-hydroxyprogesterone; A. aldosterone; A4. androstenedione; F. cortisol; E. cortisone; DHEAS. dehydroepiandrosterone sulfate; E2. estradiol; P. progesterone; T. testosterone; 11-KA4. 11-ketoandrostenedione; 11-KT. 11-ketotestosterone; 11-OHA4. 11β-hydroxyandrostenedione; 11-OHT. 11β-hydroxytestosterone;

Table S5. Quantifier and qualifier mass transitions for the 11-OAs including their IS.

Analyte	Precursor $[M+H^+]^+$ [u]	Quantifier [u]	Qualifier [u]
11-KA4	301.2	257.2	121.0
11-KA4-d10	311.2	265.3	
11-KT	303.2	259.2	121.1
11-KT-d3	306.2	262.2	
11-OHA4	303.2	267.2	145.2
11-OHA4-d7	310.2	128.1	
11-OHT	305.2	269.2	121.0
11-OHT-d5	310.2	125.1	

Table S6. Mean levels and standard derivation of 11-OAs and 17-OHP. A4. E. DHEAS. P and T as well as significant ratios in 42 CAH patients and 42 healthy individuals matched for sex and age. *p < 0.01. ***p* < 0.05. *** *p* < 0.001.

**Analyte**	**CAH (N=42)**		**Control (N=42)**		**CAH vs. Control**	
	Mean	SD	Mean	SD	p-value	
17-OHP [nmol/l]	142	203	1.3	1.1	< 0.001	***
A4 [nmol/l]	14	29	1.6	1.5	< 0.001	**
E [nmol/l]	42	25	62	21	< 0.001	***
DHEAS [µmol/l]	1208	1327	2286	2130	0.012	*
P [nmol/l]	6.3	7.5	2.2	6.1	< 0.001	***
T [nmol/l]	4.4	6.5	3.8	7.3	0.015	*
11-KA4 [nmol/l]	2.0	3.6	0.41	0.45	0.016	*
11-KT [nmol/l]	4.3	6.6	0.78	0.59	0.001	*
11-OHA4 [nmol/l]	15	27	2.1	1.8	0.006	*
11-OHT [nmol/l]	1.4	2.9	0.25	0.25	0.080	
17-OHP/11-KA4	118	118	4.9	4.6	< 0.001	***
17-OHP/11-KT	46	43	2.2	1.8	< 0.001	***
17-OHP/11-OHA4	21	22	0.89	0.70	< 0.001	***
17-OHP/11-OHT	346	619	8.3	8.2	< 0.001	***
A4/11-OHT	18	19	7.9	7.0	0.004	**
DHEAS/KA	3444	5950	7154	7081	< 0.001	***
DHEAS/KT	1236	2115	2969	2408	< 0.001	***
DHEAS/OHA	470	730	1390	1533	< 0.001	***
DHEAS/OHT	6221	8895	13524	14144	< 0.001	***
T/11-OHT	8.3	12	24	50	0.042	*
11-OHA4/11-OHT	15	13	10	5.7	0.013	*

17-OHP. 17α-hydroxyprogesterone; A4. androstenedione; E. cortisone; DHEAS. dehydroepiandrosterone sulfate; P. progesterone; T. testosterone; 11-KA4. 11-ketoandrostenedione; 11-KT. 11-ketotestosterone; 11-OHA4. 11β-hydroxyandrostenedione; 11-OHT. 11β-hydroxytestosterone; SD. standard derivation

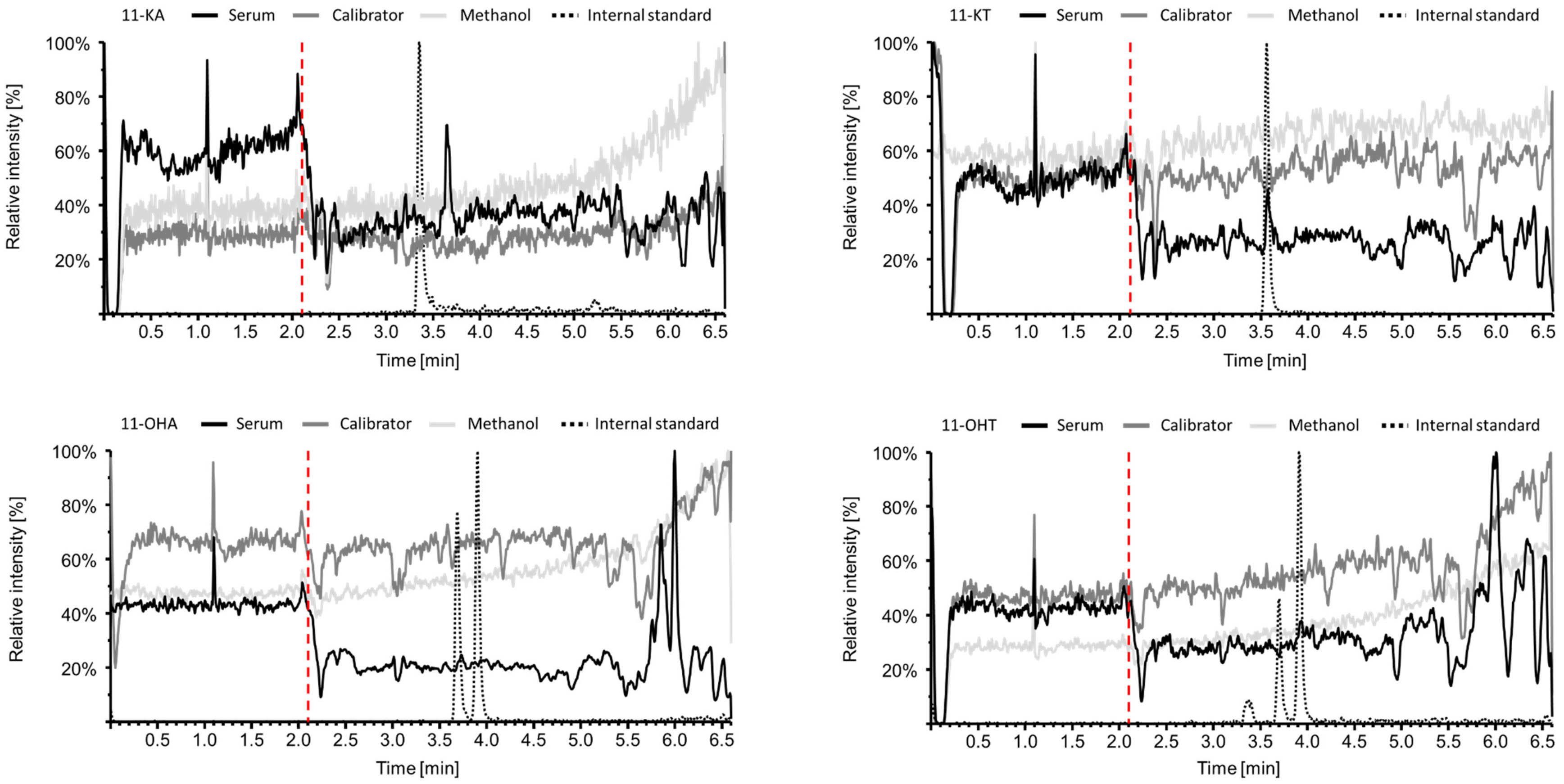

Figure S1. Exemplary chromatograms of post column infusion of four 11-OA including their corresponding internal standard (grey dashed line). In methanol (light grey) and calibrator (grey), negligible matrix effects were observed. In serum samples (black), an intensity loss by the first fraction of matrix constituents reaching the mass spectrometer was observed starting with the red dashed line in example chromatograms for 11-OAs. This ion suppression by the matrix of serum samples was observed across the detection window affecting all mass transitions of all analytes and internal standards. 11-KA4. 11-ketoandrostenedione; 11-KT. 11-ketotestosterone; 11-OHA4. 11β-hydroxyandrostenedione; 11-OHT. 11β-hydroxytestosterone.
